# Incidence and predictors of delirium on the intensive care unit in patients with acute kidney injury, insight from a retrospective registry

**DOI:** 10.1038/s41598-021-96839-x

**Published:** 2021-08-26

**Authors:** Markus Jäckel, Nico Aicher, Jonathan Rilinger, Xavier Bemtgen, Eugen Widmeier, Tobias Wengenmayer, Daniel Duerschmied, Paul Marc Biever, Peter Stachon, Christoph Bode, Dawid Leander Staudacher

**Affiliations:** 1 Department of Cardiology and Angiology I, Faculty of Medicine, Heart Center Freiburg University, University of Freiburg, Hugstetter Strasse 55, 79106 Freiburg, Germany; 2 Department of Medicine III (Interdisciplinary Medical Intensive Care), Faculty of Medicine, Medical Center, University of Freiburg, Freiburg, Germany; 3 Department of Nephrology, Faculty of Medicine, University Hospital Freiburg, University of Freiburg, Freiburg, Germany

**Keywords:** Nephrology, Neurology, Neurological disorders

## Abstract

Acute kidney injury (AKI) and delirium are common complications on the intensive care unit (ICU). Few is known about the association of AKI and delirium, as well as about incidence and predictors of delirium in patients with AKI. In this retrospective study, all patients with AKI, as defined by the KDIGO (kidney disease improving global outcome) guideline, treated for more than 24 h on the ICU in an university hospital in 2019 were included and analyzed. Delirium was defined by a NuDesc (Nursing Delirium screening scale) ≥ 2, which is evaluated three times a day in every patient on our ICU as part of daily routine. A total of 383/919 (41.7%) patients developed an AKI during the ICU stay. Delirium was detected in 230/383 (60.1%) patients with AKI. Independent predictors of delirium were: age, psychiatric disease, alcohol abuse, mechanical ventilation, severe shock, and AKI stage II/III (all p < 0.05). The primary cause of illness had no influence on the onset of delirium. Among patients with AKI, the duration of the ICU stay correlated with higher stages of AKI and the presence of delirium (stage I/no delirium: median 1.9 (interquartile range (25th–75th) 1.3–2.9) days; stage II/III/no delirium: 2.6 (1.6–5.5) days; stage I/delirium: 4.1 (2.5–14.3) days; stage II/III/delirium: 6.8 (3.5–11.9) days; all p < 0.01). Delirium, defined as NuDesc ≥ 2 is frequent in patients with AKI on an ICU and independently predicted by higher stages of AKI.

## Introduction

Delirium is a common yet major health care problem, highlighted by the costs attributed to delirium being over $182 billion in 18 European countries combined, as evaluated for 2011^1^. As delirium is notoriously underdiagnosed, delirium guidelines recommend screening for delirium with a valid and reliable delirium-score^2^. Detection of delirium is important, since it has been suggested that delirium might be preventable in 30–40% of cases^3,4^. Studies on delirium report a high incidence of delirium in specific subgroups, including critically ill patients on intensive care units (ICU), in elderly patients, or those with psychiatric diseases^5^.

While epidemiologic data suggests an association between chronic kidney disease (CKD) and development of neuropsychiatric disease (including delirium), less is known for the association of acute kidney injury (AKI) and delirium^6^. AKI is a common complication in up to 50% of all critical ill patients treated on the ICU. Furthermore, AKI is an independent predictor of short- and long-term morbidity and mortality^7,8^. Experimental data from mice showed that AKI causes inflammation and functional changes in the brain, potentially triggering acute brain dysfunction and delirium^9^. Some clinical data exists suggesting an association between AKI and delirium, when comparing patients with and without AKI^10–12^. To date, no study exists differentiating the association of delirium and the AKI stage. Defining this patients cohort might be of special interest, since incidence and predictors of delirium differ significantly in various patient collectives therefore hampering generalization^13–15^.

In this retrospective study, we analyzed all patients on the ICU with AKI and with or without delirium, as detected by the Nursing Delirium screening scale (NuDesc), to investigate delirium incidence and potential risk factors.

## Methods

We retrospectively analyzed all medical records of patients treated on the medical ICU at the University Heart Center Freiburg between 01.01.2019 and 12.31.2019. Analysis was blinded to patient identity and was covered by an ethics approval (Ethics Committee of the University of Freiburg, file number 387/19). All methods were performed in accordance with the relevant guidelines and regulations. Since only retrospective data was collected, the informed consent was waived by the ethics committee (file number 387/19).

### Patient selection and data collection

All consecutive patients treated on the ICU were included in this research and screened by a manual case-by-case review. As for exclusion criteria, patients with an ICU stay shorter than 24 h were sorted out. Also, patients with end stage renal disease on renal replacement therapy (RRT) were excluded. If delirium evaluation was not possible, patients were excluded as well (including patients with transfer to other hospitals or death before extubation, patients with severe neurologic comorbidities or hypoxic brain dysfunction). Only the first ICU stay in 2019 was analyzed excluding data from subsequent admissions.

All outcome variables were evaluated by manual case-by-case review of medical and patient records. Since only data from the index hospital stay was evaluated, no patients were lost to a follow up. Registry was checked for data integrity and plausibility according to the RECORD recommendations for data clearing^16^. Some data (like laboratory tests) were not available for all patients at all time points. In case of missing values, number of data available is given for every data point in the tables.

### Definitions

Acute kidney injury (AKI) was defined as an absolute increase of serum creatinine of ≥ 0.3 mg/dl or relatively ≥ 1.5 times of baseline creatinine, according to the KDIGO (Kidney Disease Improving Global Outcome) guideline^17^. Stages were defined as follows: stage I: creatinine 1.5–1.9 times baseline or increase ≥ 0.3 mg/dl; stage II: 2.0–2.9 times baseline; stage III: ≥ 3.0 times baseline or increase in serum creatinine to ≥ 4.0 mg/dl (with an acute increase of ≥ 0.5 mg/dl) or initiation of RRT. According to local standards, occurrence and stage of AKI are well documented in the medical records. To identify undocumented AKI, all creatinine values were recorded and AKI was classified according to the definition given above. As ICU patients were investigated, a baseline creatinine could not be reliably determined for patients with creatinine peak at admission. If no CKD and no baseline creatinine were documented in the medical records, a normal kidney function was assumed and AKI staging was performed using an age-appropriate creatinine value. In case of conflicting data from medical records and lab tests, patients were adjudicated on a case-by-case basis. Transient AKI was defined as a return to the baseline-creatinine (± 20%) within 48 h after AKI onset.

Chronic kidney disease (CKD) was defined by the baseline GFR. A documented baseline eGFR < 60 ml/min (CKD stage ≥ III KDIGO (Kidney Disease: Improving Global Outcomes) estimated with the MDRD (Modification of Diet in Renal Disease) GFR equation) or a documented CKD stage ≥ III were defined as chronic kidney disease for this research.

Severe shock was predefined as a norepinephrine dose of ≥ 1 mg over at least 4 h according to the definition used by Russel et al.^18^. In order to consider also patients with cardiogenic shock, patients requiring two different catecholamines for more than 4 h were also considered having severe shock. Primary cause of illness was adjudicated on a case-by-case basis in “cardiac” (acute myocardial infarction, transcatheter aortic valve implantation, resuscitation, cardiogenic shock, heart rhythm disturbances, pulmonary embolism), “respiratory” (pneumonia, acute respiratory distress syndrome, exacerbated chronic obstructive bronchitis, pneumothorax), “infectious” (pneumonia, urosepsis, sepsis with other focus) or “other” (hyponatremia, gastrointestinal bleeding, vasculitis, ketoacidosis, liver failure).

### Definition of delirium

Delirium is routinely assessed by specially trained nurses in all patients on the ICU at least three times a day using the NuDesc and the RASS (Richmond agitation and sedation scale) score as described before^14,15^. We established superusers, which introduced and trained the nurses in using the score. In doubt, a superuser could be consulted. The NuDesc is approved, easy to use and has a reported sensitivity (93–98%) and specificity (81–87%) for diagnosis^19–21^. Delirium was defined by NuDesc ≥ 2 in at least one assessment. To further classify delirium, the RASS score was used^22^. We defined “hyperactive delirium” as RASS ≥ 1 and no RASS < 0 in follow-up scores. RASS scores < 0 after necessary sedation due to agitation were excluded. “Hypoactive delirium” was defined as RASS ≤ 0, whereas “mixed delirium” was defined as variable positive and negative RASS. “Delirium duration” was defined as number of days with at least one NuDesc ≥ 2.

### Bias

Bias was reduced by predefining the primary endpoint “delirium” using a well-established score. Group allocation was performed after data collection thereby reducing bias. Interpretation of variables was minimized and clear cutoff values were predefined. An adjustment for confounders was done by multivariable logistic regression analysis.

### Statistical methods

All relevant data is given in standardized tables. For data analysis, SPSS (version 26, IBM Statistics) and Prism (version 8, GraphPad) were employed. For statistical analysis, Mann–Whitney U-test was used for analysis of continuous variables. For categorical variables, Fisher's exact test was used when number of expected values was smaller than five, otherwise Pearson’s Chi-squared test was performed. For comparison of length of stay, a time-to-event analysis was performed using the Log-rank (Mantel-Cox) test. A p-value of < 0.05 was considered statistically significant. Data is given as n (%), median and interquartile range (25th–75th) or odds ratio (OR) with 95% confidence interval (CI) if not stated otherwise. Two endpoints were further investigated by multivariable regression analysis. Firstly, predictors of delirium were evaluated for interactions and in order to estimate the impact on delirium development using a binary regression analysis. We incorporated only well-established predictors of delirium which are known to significantly differentiate between patients with and without delirium (forward selection process with a p-value threshold of 0.01), see Table 3 (age, preexisting psychiatric disease, alcohol abuse, mechanical ventilation, severe shock, lactate) and presence of AKI (stage II/III) into the analysis. As surrogate of infection, leukocytosis was used.

The duration of the ICU stay was correlated with well-established predictors of duration of ICU stay. A linear regression analysis was performed incorporating the same factors as used for predictors of delirium described above expanded only by delirium.

### Ethics approval and consent to participate

This retrospective study was approved by the ethics committee of the Albert Ludwigs University of Freiburg, file number 387/19. Informed consent was waived by the approval of the relevant ethic committee (Ethics Committee of Albert Ludwigs University of Freiburg, file number 387/19).

## Results

### Patient collective

In 2019, 1039 patients were treated on our medical ICU for more than 24 h. Of these, 120 patients were excluded. 80 patients died or were transferred to other hospitals before extubation, 16 had severe neurologic comorbidities or hypoxic brain dysfunction and 24 were on chronic renal replacement therapy, leading to 919 patients who were analyzed for AKI.

**Figure 1:**
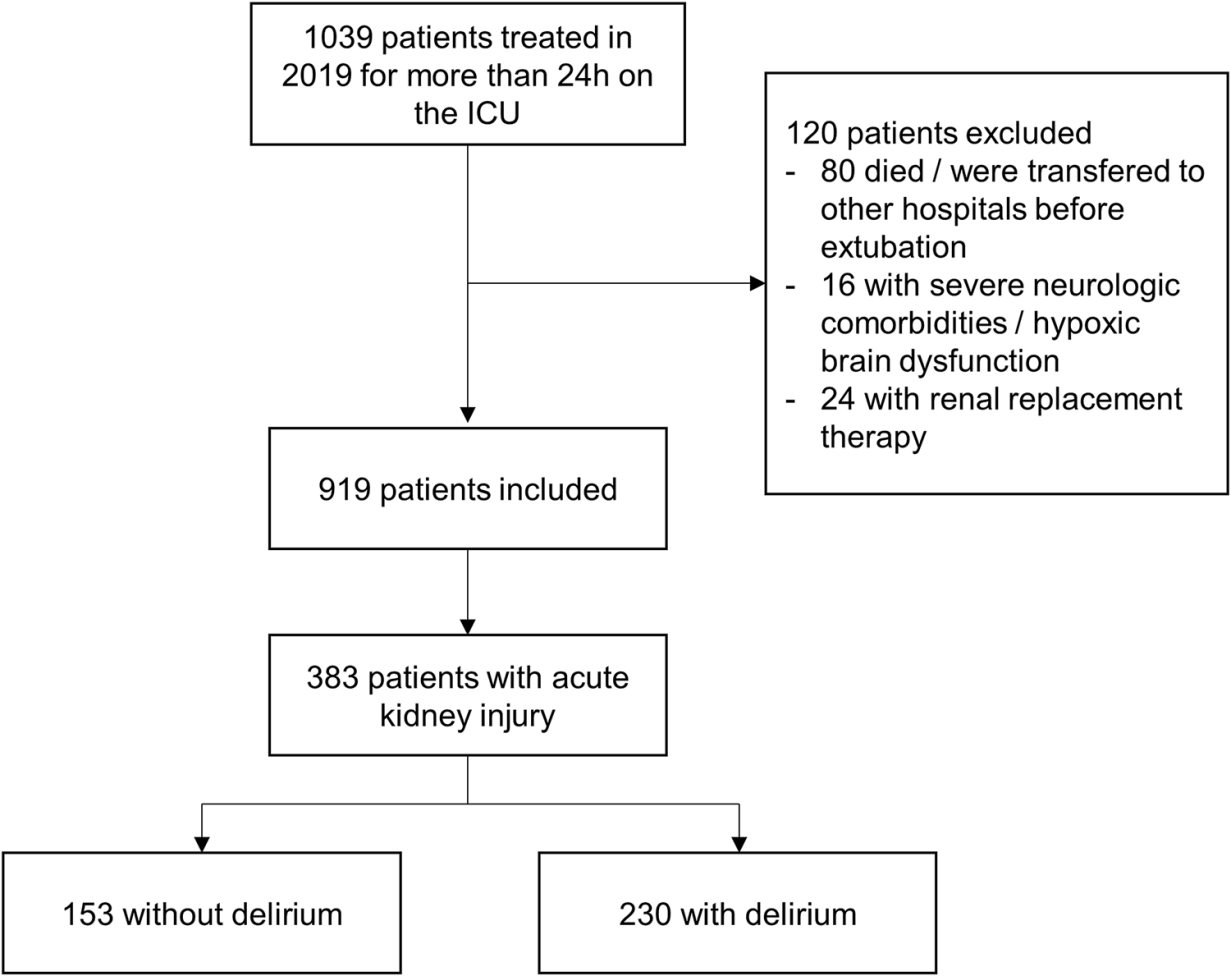
Flowchart indicating number of included and excluded patients. Data are given as number of patients.

Of 919 patients, 383 (41.6%) patients had AKI (Fig. 1). Median age of patients with AKI was 70.1 (59.9–79.1) years and 32.1% were female. AKI stage I was detected in 196/383 (51.2%), stage II in 57/383 (14.9%) and stage III in 130/383 (33.9%) patients. RRT was necessary in 52/383 (13.6%) patients. Primary cause of illness was cardiac in 175/383 (45.7%), respiratory in 75/383 (19.6%), and infectious in 97/383 (25.3%) patients. In 70/383 (18.3%) patients, treatment on our ICU occurred for other reasons.

### Delirium

Delirium was detected in 230/383 (60.1%) patients with AKI. While 47/230 (20.4%) subjects had hyperactive delirium, 79/230 (34.3%) presented in hypoactive and 104/230 (45.2%) in mixed delirium. Median highest NuDesc reported was 4 (3–5), median duration of delirium was 3 (1–5) days. In 90% of all patients, delirium started within the first 5 days after admission with a median of 1 (0–3) days (Table 1). Patients with delirium were significantly older than patients without delirium (73.2 versus 68.1 years, p = 0.001) and had more preexisting neurological- and psychiatric diseases (Table 2).

**Table 1:** Delirium characteristics.

**Delirium charcteristics**	
Duration (days)	3 (1–5)
Hypoactive	79 (34.3%)
Hyperactive	47 (20.4%)
Mixed	104 (45.2%)
NuDesc score at maximum	4 (3–5)
Onset of delirium (day)	1 (0–3)

p value reported in bold if difference is significant (p < 0.05). Data are given as median and interquartile range (25th-75th) or number of patients (percent of all patients).

**Table 2:** Baseline characteristics of all patients with acute kidney injury.

Baseline characteristics	No delirium (N = 153)	Delirium (N = 230)	p-value
Age	68.1 (56.9–76.0)	73.2 (62.4–80.1)	**0.001**
Female	49 (32.0%)	74 (32.2%)	0.976
**Comorbidities**			
Coronary heart disease	40 (26.1%)	68 (29.6%)	0.466
Heart rhythm disturbances	30 (19.6%)	87 (37.8%)	** < 0.001**
Obesity	24 (15.7%)	36 (15.7%)	0.993
Pulmonary disease	31 (20.3%)	44 (19.1%)	0.785
Liver disease	15 (9.8%)	28 (12.2%)	0.472
Chronic kidney disease	55 (35.9%)	87 (37.8%)	0.709
Peripherial/cerebral arterial occlusive disease	16 (10.5%)	32 (13.9%)	0.317
Neurological disease	23 (15.0%)	62 (27.0%)	**0.006**
Malignancy	33 (21.6%)	37 (16.1%)	0.174
Psychiatric disease/dementia	6 (3.9%)	41 (17.8%)	** < 0.001**
Alcohol abuse	6 (3.9%)	25 (10.9%)	**0.015**
Drug abuse	2 (1.3%)	9 (3.9%)	0.135

p value reported in bold if difference is significant (p < 0.05). Data are given as median and interquartile range (25th–75th) or number of patients (percent of all patients in group).

The ICU stay was significantly longer in patients with delirium (5.7 (2.7–9.9) versus 2.0 (1.3–3.6) days, p < 0.001). Patients with delirium were more often on mechanical ventilation, necessitated more catecholamine therapy and sedation, and had more frequently a central venous catheter, peripheral arterial catheter, and urinary catheter (all p < 0.001). Transient AKI was detected at similar rates in patients with and without delirium. No significant differences were detected concerning the primary cause of illness. Highest lactate value (“lactate at maximum”) was higher and leukocytosis occurred more often in patients with delirium (both p < 0.01, see Table 3). For further laboratory tests, see Supplemental Table S1.

**Table 3:** Clinical characteristics of all patients with acute kidney injury.

Clinical characteristics	No delirium (N = 153)	Delirium (N = 230)	p-value
ICU stay (days)	2.0 (1.3–3.6)	5.7 (2.7–9.9)	** < 0.001**
Mortality	12 (7.8%)	50 (21.7%)	** < 0.001**
TISS 10	6 (5–13); N = 150	10 (5–15); N = 229	** < 0.001**
SAPS 2	40 (32–50.3); N = 150	48 (40–56); N = 229	** < 0.001**
**Cause of illness**			
Cardiac	75 (49.0%)	100 (43.5%)	0.286
Respiratory	24 (15.7%)	51 (22.2%)	0.117
Infectious	34 (22.2%)	63 (27.4%)	0.255
Other	27 (17.6%)	43 (18.7%)	0.795
Resuscitation	3 (2.0%)	28 (12.6%)	** < 0.001**
Non-invasive ventilation	33 (21.6%)	101 (43.9%)	** < 0.001**
Invasive mechanical ventilation	29 (19.0%)	92 (40.0%)	** < 0.001**
Days on mechanical ventilation	0 (0–0.3)	0.5 (0–4.8)	** < 0.001**
Catecholamine therapy (shock)	41 (33.3%)	158 (68.7%)	** < 0.001**
Severe shock	24 (15.7%)	93 (40.4%)	** < 0.001**
ECMO	4 (2.6%)	18 (7.8%)	**0.032**
Opiate	86 (56.2%)	172 (74.8%)	** < 0.001**
Propofol	38 (24.8%)	127 (55.2%)	** < 0.001**
Isoflurane	1 (0.7%)	27 (11.7%)	** < 0.001**
α2-adrenoceptor agonist	15 (9.8%)	105 (45.7%)	** < 0.001**
Coronary angiography	36 (23.5%)	57 (24.8%)	0.779
AKI stage II/III	62 (40.5%)	125 (54.3%)	**0.008**
Renal replacement therapy	18 (11.8%)	34 (14.8%)	0.398
Transient AKI	35 (22.9%)	38 (16.5%)	0.121
Arterial line	97 (63.4%)	196 (85.2%)	** < 0.001**
Central venous catheter	77 (50.3%)	168 (73.0%)	** < 0.001**
Urinal catheter	96 (62.7%)	216 (93.9%)	** < 0.001**
Necessity of blood transfusion	45 (29.4%)	95 (41.3%)	**0.018**
Lactate at maximum mmol/l	2.5 (1.7–4.2)	3.6 (2.2–5.3); N = 229	** < 0.001**
Leucocytosis (> 10^4^/µl)	105 (68.6%)	192 (83.5%)	**0.001**

p value reported in bold if difference is significant (p < 0.05). Data are given as median and interquartile range (25th–75th) or number of patients (percent of all patients in group).

### Delirium and AKI stage

**Figure 2:**
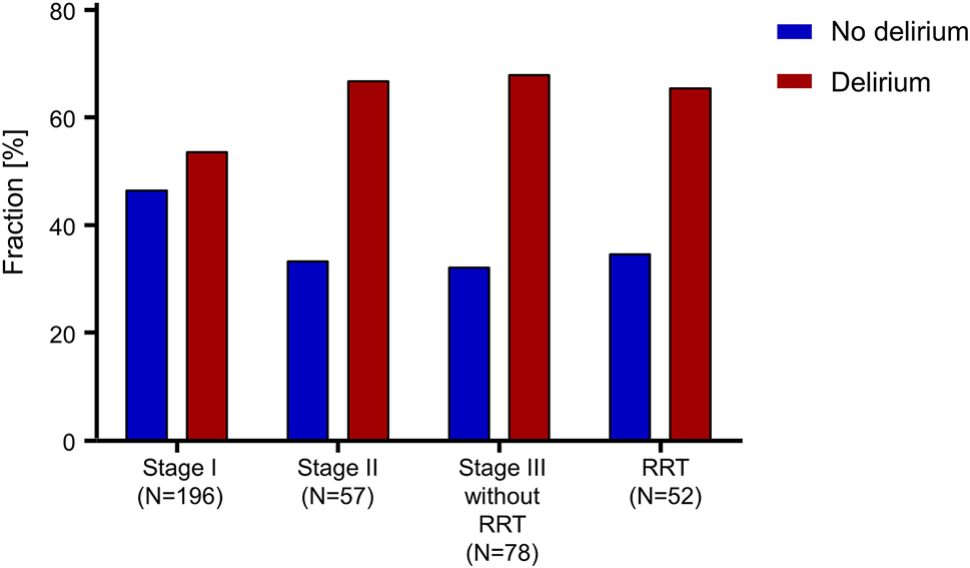
Delirium incidence and stage of acute kidney injury. Graph shows distribution of delirium positive patients per stage of acute kidney injury. Stage III was separated in patients with and without renal replacement therapy (RRT).

Of all patients with AKI stage I, 105/196 (53.6%) had delirium. Delirium rate increased in patients with stage II (38/57; 66.7%). No further increase was detected in stage III (87/130; 66.9%) or in the subgroup of stage III receiving RRT (34/52; 65.4%) (stage I versus stage II/III, p = 0.008) (Fig. 2).

### Predictors of delirium

**Figure 3:**
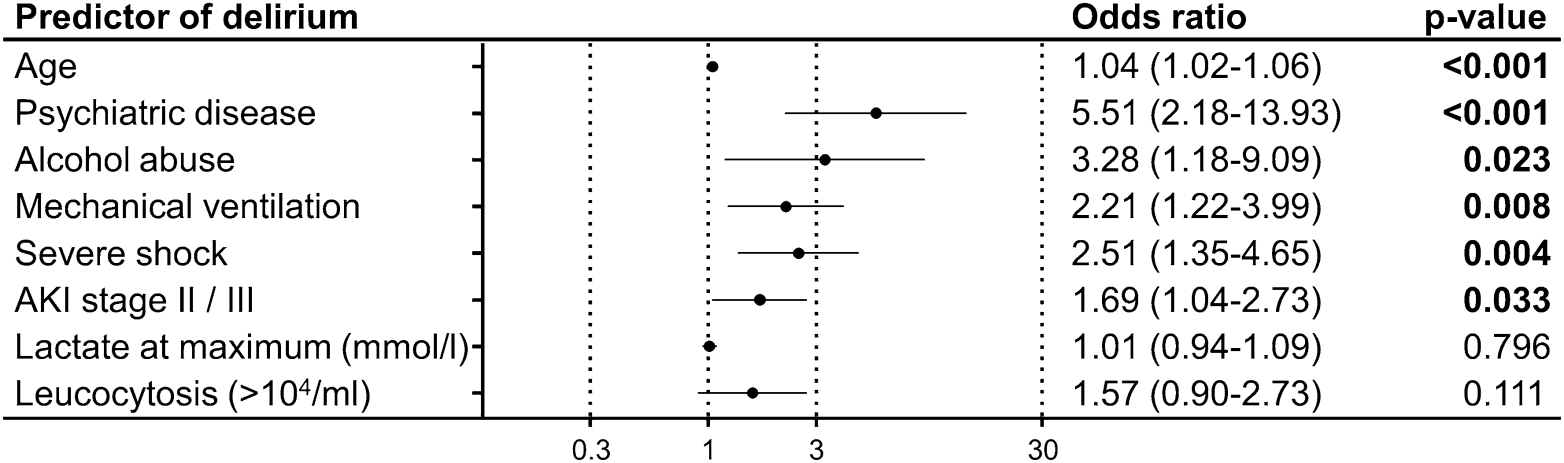
Predictors of delirium on the ICU in patients with acute kidney injury. Graph shows multivariable binary logistic regression analysis with odds ratio (95% confidence interval) of different predictors for delirium in patients with acute kidney injury (AKI) staying for more than 24 h on the ICU.

Multivariable binary logistic regression analysis was performed to identify independent predictors of delirium in patients with AKI. Independent predictors were age (1.04 (1.02–1.06, p < 0.001), psychiatric diseases (5.51 (2.18–13.93), p < 0.001), alcohol abuse (3.28 (1.18–9.09), p = 0.023), severe shock (2.51 (1.35–4.65)), p = 0.004), invasive ventilation (2.21 (1.22–3.99, p = 0.008) and stage II/III of AKI (1.69 (1.04–2.73), p = 0.033), while laboratory markers as lactate and leukocytosis were no independent predictors for delirium (Fig. 3).

### Outcome with delirium

**Figure 4:**
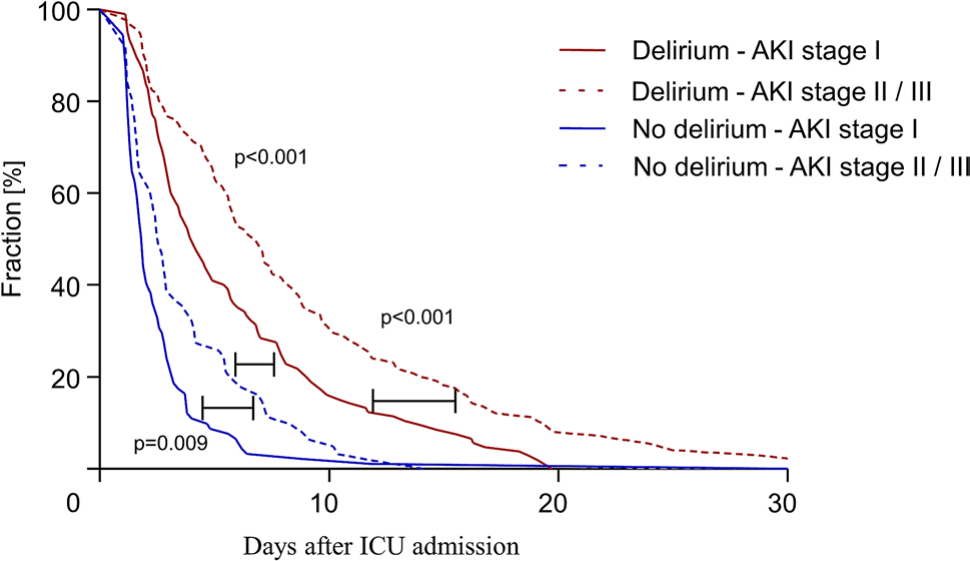
Duration of stay on the ICU. Graph showing ICU stay of all patients with acute kidney injury (AKI) staying for more than 24 h on the ICU separated by stage of AKI and incidence of delirium.

When analyzing the duration of the ICU stay, patients with AKI stage I without delirium had the shortest duration (1.9 (1.3–2.9) days), followed by patients with AKI stage II/III without delirium (2.6 (1.6–5.5) days) and patients with AKI stage I with delirium (4.1 (2.5–14.3) days). Patients with AKI stage II/III and delirium had the longest duration of the ICU stay (6.8 (3.5–11.9) days; all p < 0.01) (Fig. 4).

Multivariable linear regression analysis showed that delirium, as well as a higher stage of AKI (stage II/III) are independently associated with a longer ICU stay (delirium: coefficient 2.53 (1.37–3.68), p < 0.001; AKI stage II/III: coefficient 1.44 (0.37–2.51), p = 0.009), see Supplemental Fig. S1.

## Discussion

In this retrospective registry, we report a high incidence of delirium in patients with acute kidney injury (AKI). After adjusting for numerous confounders, we found that among patients with AKI, higher stages of AKI (stage II/III) are independently associated with delirium. Furthermore, higher stages of AKI (stage II/III) as well as delirium are independently associated with a longer ICU stay in patients with AKI.

We found delirium in 60.1% of patients with AKI. This is in concordance with the incidence of delirium reported in other ICU cohorts, ranging from 22 to 83% depending on assessment methods and population^14,15,23,24^. While the incidence was lower in AKI stage I (53.6%), the incidence increased in stage II (66.7%) and stage III (66.9%). There are several pathophysiological explanations for an increased risk of delirium in higher stages of AKI. Multiple uremic toxins are involved in the uremic encephalopathy^25–27^. Disturbance of the blood–brain barrier has also been shown to affect acute and chronic uremic encephalopathy^28^. Furthermore, AKI induces inflammation and functional changes in the brain in an experimental model^9^. Few data on delirium in patients with AKI is available, mostly focusing on the comparison of patients with AKI to those without^10–12^. Siew et al. showed that higher stages of AKI are risk factors for delirium and coma in critically ill patients^10^. Therefore, our data is in concordance to literature and shows an association of higher stages of AKI (stage II/III) and delirium in medical patients when compared to patients with AKI stage I. Furthermore, we were able to adjust for confounders of delirium thereby reducing bias. Interestingly, transient AKI was not detected more frequently in patient with delirium compared to those without—potentially indicating that patients with transient AKI may not have an increased risk of delirium when compared to patients with non-transient AKI.

It has been shown that AKI stage III is a risk factor of hyperactive delirium^12^. Our data furthermore suggests that AKI stage II/III is associated with all types of delirium (hyperactive, hypoactive, and mixed) when compared to AKI stage I. Results presented suggest an independent association of AKI stage, delirium, and the duration of ICU stay. Patients with AKI stage I without delirium had the shortest duration of ICU stay opposed to patients with AKI stage II/III and delirium with longest durations of ICU stay. This might help identifying a patient collective requiring special attention. As primary prevention of delirium with nonpharmacologic multicomponent approaches is the most effective delirium therapy^1^, special attention should be given to patients with higher stages of AKI.

Incidence of delirium in patients requiring RRT was similar to patients with AKI stage II/III without RRT. Limited data on RRT and delirium show that RRT might modify the association between AKI and acute brain dysfunction thereby attenuating the effects of AKI on the brain (by removing sedatives, antibiotics or other metabolites)^10,29–31^. Since numbers of patient on RRT are limited in our registry, we were not able to investigate this hypothesis in our data. Future studies might clarify whether RRT affects the severity or duration of delirium in patients with AKI.

Most literature on delirium focuses on specific patient cohorts such as mechanically ventilated patients, elderly or surgical patients when investigating delirium^32,33^. In these patients, common predictors for delirium are age, shock, alcohol misuse, and mechanical ventilation^1,12,15,34,35^. We could confirm these independent predictors of delirium in patients with AKI. Furthermore, psychiatric diseases (including dementia) are the most consistently observed risk factors for delirium^13,36–39^ in literature and in our registry. Interestingly, delirium incidence was not significantly associated with the primary cause of illness when summarized in the categories used in this research. In our patient collective, cause for admission seems to play a minor role.

### Limitations

When generalizing the results presented in this study, some limitations have to be considered. Firstly, there are several delirium screening scores used in literature^21^. In this retrospective study we used the NuDesc to define delirium. The NuDesc requires less than one minute per patient and is easy to use, whereas other scores such as the CAM-ICU and ICDSD require special instructions and are significantly more time consuming^19^. Although sensitivity and specificity reported previously are high (93–98% and 81–87%, respectively), our definition of delirium is not congruent to the DSM-5 criteria^19–21^. Secondly, due to the retrospective nature of our study, baseline kidney function was not determinable in all cases. For patients with no documented stage of AKI and no documented CKD in the medical records, a normal baseline kidney function was assumed. Although on our ICU medical records are routinely requested, we cannot rule out the possibility that patients were assessed as having acute kidney failure when they actually had undetected chronic kidney disease. Additionally, we did not record urine output in our patients and therefore could have potentially missed patients with early stages of AKI and normal serum creatinine values.

We present retrospective data from a single-center. Consequently, results should be considered hypothesis-generating only and should be confirmed in larger multi-center studies. Clinical data was based on medical reports. Because we did not use structured clinical interviews, some variables, as alcohol abuse, may be underestimated. In addition, because of the retrospective nature, we probably did not capture all ICU complications that may have facilitated delirium.

## Conclusion

In a cohort of patients with AKI on the ICU, a NuDesc ≥ 2 is an independent predictor of ICU stay. Delirium, defined as a NuDesc ≥ 2, is frequent and independently predicted by AKI stage II/III. Since causality however cannot be proven by retrospective data, prospective studies are required to validate findings and further investigate the mechanism by which AKI leads to delirium.

## Supplementary Information

Supplementary Figure S1.

Supplementary Table S1.

## Data Availability

The datasets used and analyzed during the current study are available from the corresponding author on reasonable request.

## Abbreviations

AKI: Acute kidney injury
ICU: Intensive care unit
KDIGO: Kidney disease improving global outcome
NuDesc: Nursing delirium screening scale
RASS: Richmond agitation and sedation scale
RRT: Renal replacement therapy
