# Supplementary figures and images for: Incidence and predictors of delirium on the intensive care unit in patients with acute kidney injury, insight from a retrospective registry
Source: Sci Rep. 2021 Aug 26;11:17260. doi: 10.1038/s41598-021-96839-x (PMC8390667; doi:10.1038/s41598-021-96839-x)

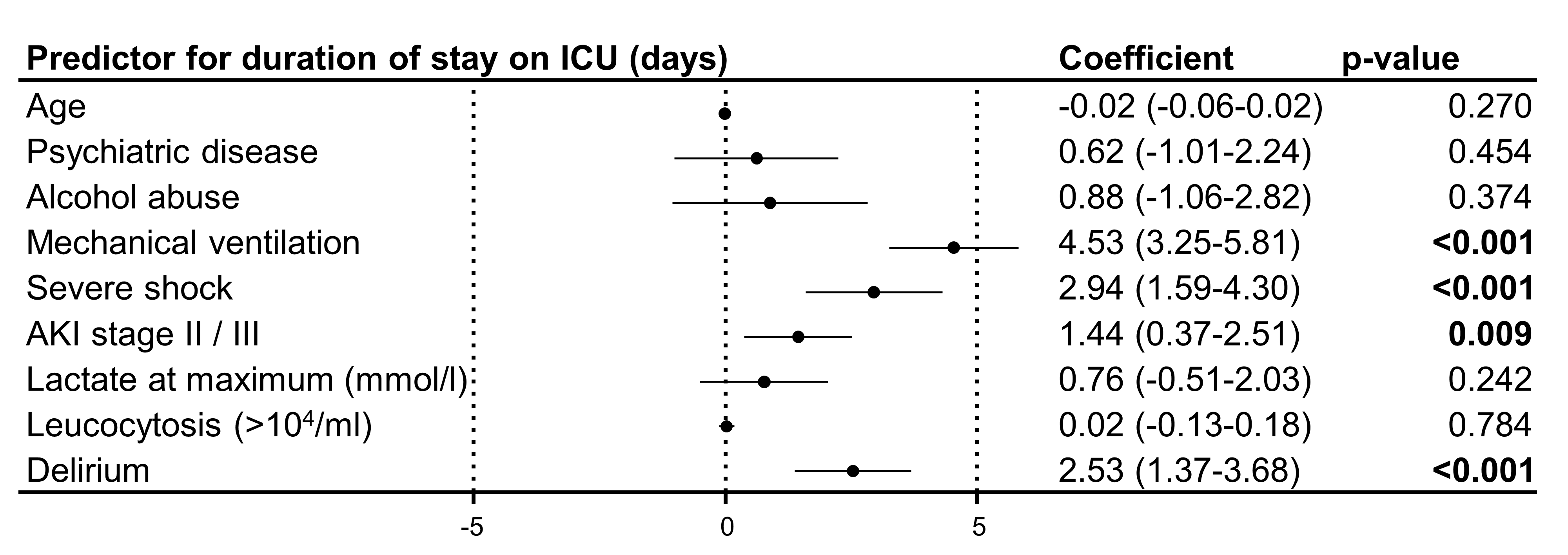

Supplement: Supplementary file 1 — Supplementary Figure S1. [file 41598_2021_96839_MOESM1_ESM.tif]
