# Supplementary material for: Incidence and predictors of delirium on the intensive care unit in patients with acute kidney injury, insight from a retrospective registry
Source: Sci Rep. 2021 Aug 26;11:17260. doi: 10.1038/s41598-021-96839-x (PMC8390667; doi:10.1038/s41598-021-96839-x)
Supplement: Supplementary file 2 — Supplementary Table S1. [file 41598_2021_96839_MOESM2_ESM.docx]

**Incidence and predictors of delirium on the intensive care unit in patients with acute kidney injury, insight from a retrospective registry**

**Markus Jäckel^1,2^, Nico Aicher^1,2^, Jonathan Rilinger^1,2^, Xavier Bemtgen^1,2^, Eugen Widmeier^2,3^, Tobias Wengenmayer^1,2^, Daniel Duerschmied^1,2^, Paul Marc Biever^1,2^, Peter Stachon^1,2^, Christoph Bode^1,2^, Dawid Leander Staudacher^1,2^**

^1^Department of Cardiology and Angiology I, Heart Center Freiburg University, Faculty of Medicine, University of Freiburg, Germany

^2^Department of Medicine III (Interdisciplinary Medical Intensive Care), Medical Center, University of Freiburg, Faculty of Medicine, University of Freiburg, Germany

³Department of Nephrology, University Hospital Freiburg, Faculty of Medicine, University of Freiburg, Germany

**Supplemental table 1** Laboratory characteristics of all patients with acute kidney injury.

p value reported in bold if difference is significant (p < 0.05). Data are given as median and interquartile range (25th-75th) or number of patients (percent of all patients in group). Maximum values were analyzed if not stated otherwise.

| **Laboratory characteristics** | **No delirium (N=153)** | **Delirium (N=230)** | **p-value** |
| --- | --- | --- | --- |
| Bilirubin mg/dl | 1.3 (0.9-1.9) | 1.5 (1.0-2.5); N=229 | **0.004** |
| Hb minimum g/dl | 9.3 (7.3-12.0) | 7.8 (6.6-9.8); N=229 | **<0.001** |
| pH minimum | 7.32 (7.21-7.38) | 7.22 (7.15-7.32); N=229 | **<0.001** |
| CK U/l | 199 (99-500); N=124 | 321 (84-981); N=203 | **0.033** |
| CRP mg/dl | 96 (32-200); N=150 | 157 (79-279) | **<0.001** |
| Baseline-creatinine | 1.02 (0.88-1.30); N=127 | 1.00 (0.85-1.45); N=191 | 0.910 |
| Creatinine at admission mg/dl | 1.69 (1.29-2.79) | 1.91 (1.38-2.94) | 0.227 |
| Creatinine mg/dl | 1.92 (1.46-3.46) | 2.48 (1.68-3.93) | **0.004** |
| LDH U/l | 365 (251-711); N=124 | 422 (277-751); N=211 | 0.098 |
| Leucocytes *10³/µl | 13.4 (8.4-25.5); N=152 | 16.9 (11.7-23.0) | **<0.001** |
| Procalcitonin ng/ml | 1.6 (0.3-17.3); N=86 | 2.2 (0.6-15.6); N=173 | 0.214 |

**Supplemental figure 1 Predictors of duration of stay on the ICU in patients with acute kidney injury.** Graph shows multivariate linear regression analysis with regression coefficient (95% confidence interval) for predictors for duration of stay (days) on the ICU in patients with acute kidney injury (AKI) staying for more than 24 hours on the ICU
